# Therapeutic efficacy of acupuncture point stimulation for stomach cancer pain: a systematic review and meta-analysis

**DOI:** 10.3389/fneur.2024.1334657

**Published:** 2024-04-04

**Authors:** Xuancheng Zhou, Jieying Zhang, Lai Jiang, Shengke Zhang, Yuheng Gu, Jingyi Tang, Tong Pu, Xiaomin Quan, Hao Chi, Shangke Huang

**Affiliations:** ^1^Clinical Medical College, Southwest Medical University, Luzhou, China; ^2^First Teaching Hospital of Tianjin University of Traditional Chinese Medicine, Tianjin, China; ^3^National Clinical Research Center for Chinese Medicine Acupuncture and Moxibustion, Tianjin, China; ^4^College of Acupuncture and Tuina and Rehabilitation, Hunan University of Chinese Medicine, Changsha, China; ^5^Beijing University of Chinese Medicine Second Affiliated Dong Fang Hospital, Beijing, China; ^6^Department of Oncology, The Affiliated Hospital of Southwest Medical University, Luzhou, China

**Keywords:** acupuncture point stimulation, stomach cancer pain, therapeutic efficacy, traditional Chinese medicine, meta-analysis

## Abstract

**Purpose:**

In recent years, traditional Chinese medicine has received widespread attention in the field of cancer pain treatment. This meta-analysis is the first to evaluate the effectiveness and safety of acupuncture point stimulation in the treatment of stomach cancer pain.

**Methods:**

For this systematic review and meta-analysis, we searched PubMed, Web of Science, Cochrane Library, Embase, WANFANG, China National Knowledge Infrastructure (CNKI), and Chinese Journal of Science and Technology (VIP) databases as well as forward and backward citations to studies published between database creation to July 27, 2023. All randomized controlled trials (RCTs) on acupuncture point stimulation for the treatment of patients with stomach cancer pain were included without language restrictions. We assessed all outcome indicators of the included trials. The evidence from the randomized controlled trials was synthesized as the standardized mean difference (SMD) of symptom change. The quality of the evidence was assessed using the Cochrane Risk of Bias tool. This study is registered on PROSPERO under the number CRD42023457341.

**Results:**

Eleven RCTs were included. The study included 768 patients, split into 2 groups: acupuncture point stimulation treatment group (*n* = 406), medication control group (*n* = 372). The results showed that treatment was more effective in the acupuncture point stimulation treatment group than in the medication control group (efficacy rate, RR = 1.63, 95% CI 1.37 to 1.94, *p* < 0.00001), decreasing in NRS score was greater in acupuncture point stimulation treatment group than in the medication control group (SMD = −1.30, 95% CI −1.96 to −0.63, *p* < 0.001).

**Systematic Review Registration:**

https://clinicaltrials.gov/, identifier CRD42023457341.

## Background

Stomach cancer ranks as the fifth most prevalent form of cancer worldwide and stands as the third leading contributor to cancer-related mortality (1). Pain represents one of the prevailing symptoms among individuals diagnosed with cancer. Within populations afflicted by solid tumors, the collective incidence of clinically significant chronic pain varies from 15% to exceeding 75% (2). From a pathophysiological perspective, chronic cancer pain stems from two primary factors. The first is directly linked to the tumors themselves, while the second is associated with diverse anticancer interventions, including surgery, chemotherapy, and radiation therapy. Tumor expansion and pain resulting from compression constitute approximately 75% of cancer-related pain, while treatment-induced discomfort comprises about 25% of such pain. These types of pain can be further categorized as nociceptive, stemming from ongoing tissue damage, or neuropathic, arising from nerve impairment or dysfunction (3). Factors encompassing fear, anxiety, and depression can collectively contribute to heightened pain levels and occurrences of breakthrough pain (4). The World Health Organization (WHO) furnishes guidelines for the pharmacological and radiotherapeutic control of cancer pain, underscoring the judicious utilization of opioids (5). Nonetheless, the opioid crisis has exacerbated the complexities of pain management, shedding light on the necessity for nonpharmacological treatment approaches (6, 7).

Traditional Chinese medicine (TCM) has a rich history of practice and is progressively gaining broader recognition for its potential in delivering remedies and therapeutic interventions for various diseases and physiological conditions (8, 9). Acupuncture point stimulation is a general term for a class of Chinese medical therapies that have been widely used in clinical practice by intervening at acupoints, especially in alleviating perioperative pain, decreasing intraoperative stress, enhancing the body’s immunity, improving patient comfort, and decreasing the incidence of postoperative complications. There are three main acupuncture point stimulation methods in relieving cancer pain, including acupuncture, moxibustion and acupoint injection. Acupuncture, as the predominant modality within traditional Chinese medicine for physical intervention, has gained widespread application in the management of chronic pain (10). Studies have shown that acupuncture may relieve neuropathic pain by inhibiting P2X7R (11). Evidence derived from clinical trials has demonstrated the safety and efficacy of acupuncture as an adjunctive therapy for alleviating cancer-related symptoms (12, 13). Moxibustion, a traditional therapeutic practice within TCM, entails the application of ignited mugwort (Artemisia vulgaris) either directly or indirectly on acupuncture points or specific body regions, with the aim of treating or preventing various diseases (14, 15). Presently, there is a burgeoning global interest and prevalence in the practice of moxibustion (16). The therapeutic technique of acupoint injection is extensively documented and has demonstrated faster and more potent clinical outcomes compared to muscle and subcutaneous injections (17). All three acupoint-related treatments play a pivotal role in the management of pain associated with stomach cancer (18).

Before this, numerous prior meta-analyses have examined the application of TCM for cancer-related pain. However, the majority of these analyses have centered exclusively on acupuncture as a methodology and encompassed studies involving pain across diverse types of cancers (19–21). Consequently, as clinical trials continue to advance, we maintain that a more targeted meta-analysis is essential to evaluate the effectiveness of acupressure point stimulation in addressing pain associated with stomach cancer. This approach encompasses well-defined outcome measures. Furthermore, our study represents the most comprehensive meta-analysis of acupressure point stimulation therapy, encompassing all pertinent recent studies available to date. Notably, this study stands as the inaugural exploration within TCM for the treatment of stomach cancer pain.

## Methods

### Search strategy and data mining

Literature databases including PubMed, Web of Science, Cochrane Library, Embase, WANFANG, China National Knowledge Infrastructure (CNKI), and Chinese Journal of Science and Technology (VIP) were systematically searched for randomized controlled trials (RCTs) focusing on acupressure point stimulation for stomach cancer-related pain. These searches encompassed literature published from the inception of each database up to July 27, 2023, in order to retrieve pertinent research. This search process was conducted collaboratively by two authors. In instances where disparities arose during the process, the authors would engage in consultation with a senior author to arrive at a consensus. The search terms employed were confined to “acupressure point therapy,” “pain,” and “stomach cancer.” The keywords of interventions included “Acupressure point stimulation” OR “Acupressure point therapy” OR “acupuncture” OR “Acupuncture” OR “Electroacupuncture” OR “Fire needle” OR “Acupuncture point injection” OR “Acupressure points” and the keywords of disease included “stomach cancer” OR “Neoplasm, Stomach” OR “Gastric Neoplasms” OR “Neoplasm, Gastric” OR “Cancer of Stomach” OR “Stomach Cancers” OR “Cancer, Gastric” OR “Gastric Cancer, Familial Diffuse” and “pain” OR “Pain, Burning” OR “Suffering, Physical” OR “Physical Suffering” OR “Pain, Migratory” OR “Pain, Radiating” OR “Ache”. During the search procedure, the initial step involved utilizing interventions as criteria to obtain relevant research articles. Subsequently, a similar approach was employed to retrieve results related to stomach cancer pain, and the outcomes of the first and second steps were merged. All retrieved reports from various databases were imported into a citation management software (EndNote, version 20) for subsequent analysis. No particular limitations were imposed on the types of articles included. Furthermore, an exhaustive review of all pertinent previously published meta-analyses and their reference lists was conducted. It is important to note that, to the best of our knowledge, there have been no recent updates on this topic to allow for a more precise assertion regarding the absence of recent reports. Details of the search strategies were shown in Supplementary File S1.

### The selection of studies/inclusion criteria

We applied the following set of inclusion criteria during the report selection process: (1) Patients were diagnosed with “pain of stomach cancer” based on explicit diagnostic (inclusion) criteria. This criterion was irrespective of age, gender, duration, or source of cases, and patients did not have any other concurrent diseases. (2) The reports were randomized controlled trials that investigated the use of acupressure point stimulation (which includes acupuncture, acupuncture point injection, or moxibustion) as a therapeutic intervention. (3) The assessment of patients’ conditions was conducted using standardized efficacy evaluation criteria, such as Numeric Rating Scale (NRS) scores, efficacy rates, etc. Reports written in any language were eligible for inclusion. Exclusion criteria encompassed: (1) Studies involving animal experiments, (2) Repetitive experiments, (3) Studies with incomplete data (e.g., missing sections like conference abstracts), (4) Studies published prior to the year 2000, and (5) Studies in which additional treatments alongside acupressure point stimulation (e.g., acupuncture combined with traditional Chinese medicine) were applied in the treatment group.

### Quality assessment

The risk of bias inherent in the included randomized controlled trials was evaluated using the revised Cochrane Risk of Bias Tool (RoB-2). For each of the following aspects, namely random sequence generation (selection bias), allocation concealment (selection bias), blinding of participants and personnel (performance bias), blinding of outcome assessment (detection bias), incomplete outcome data (attrition bias), selective reporting (reporting bias), and other sources of bias, a determination of low, unclear (indicating some concerns), or high degree of bias was assigned. The outcomes of this bias assessment were then graphically depicted using the Revman 5.4 software.

### Statistical analysis

The outcomes, including the significantly efficient rate, efficacy rate, and adverse reactions, alongside the sample sizes of the investigated studies, were input into the Revman software for conducting meta-analysis. The results were then visualized through forest plots. The level of heterogeneity was evaluated using the I^2^ index, where values up to 30% indicated mild heterogeneity, 31–50% suggested moderate heterogeneity, and values exceeding 50% indicated substantial heterogeneity. In cases where effects displayed heterogeneity (I^2^ > 50%), a random effects model was employed for the analysis. Conversely, a fixed effects model was utilized when the data appeared to be homogeneous. The calculated outcome measures and their corresponding 95% confidence intervals (CI) were illustrated in the forest plot. To determine statistical significance, a *p*-value less than 0.05 was considered indicative. During the analysis, we categorized the data into three sub-groups based on different methods of acupuncture point stimulation, and performed subgroup meta-analysis accordingly. Additionally, for a more in-depth analysis of acupuncture points, we utilized the GEMTC package in R version 4.3.0. Sensitivity analysis of the study using a case-by-case culling approach. Publication bias was estimated with a funnel plot.

## Results

### Search results

**Figure 1:**
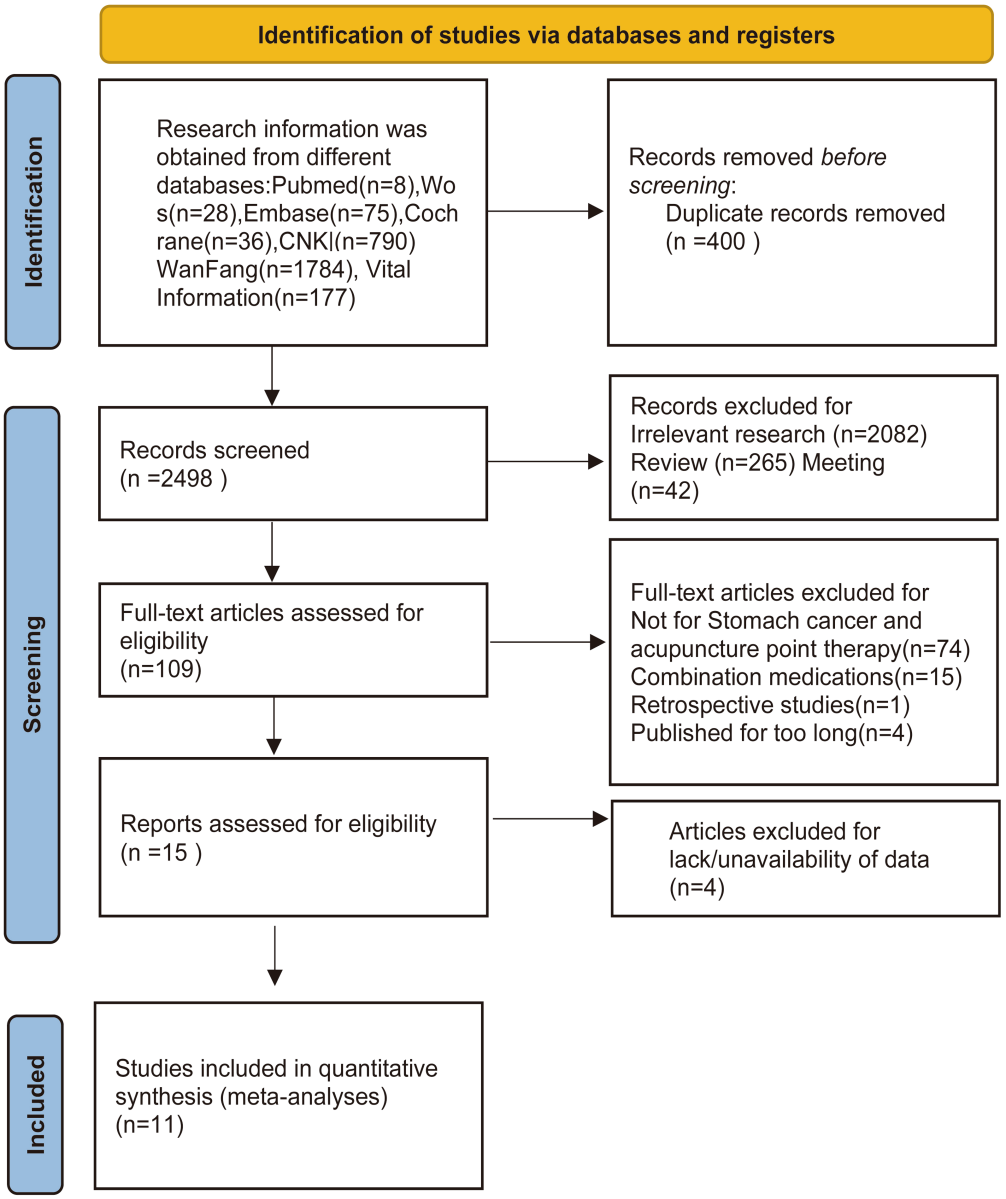
Flow diagram showing the screening and selection process of reports to be included in the meta-analysis.

At the outset, our search using the designated terms yielded a total of 585 potential research articles. Among these, 131 duplicate studies were eliminated through EndNote 20. Upon reviewing the titles and abstracts, 404 studies were identified as irrelevant and subsequently excluded. Furthermore, 307 citations were discarded due to their nature as reviews or conference materials. Subsequently, a thorough examination of the full text was conducted for 109 articles. Among these, 94 were excluded for reasons such as involving combination medications, excessive time since publication, being retrospective studies, or not being pertinent to stomach cancer and acupuncture point therapy. A meticulous evaluation of the full text was then undertaken for the remaining 15 citations. In this phase, an additional 4 studies were excluded due to insufficient data. Ultimately, after careful scrutiny, a total of 11 clinical studies met the criteria and were deemed suitable for inclusion in the meta-analysis (22–32) (Figure 1).

### Characteristics of the included studies

The studies incorporated in Table 1, from which the clinical data were extracted, were published within the time frame spanning 2002 to 2022. All of these studies were conducted within China. The collective of 11 randomized controlled trials encompassed a total of 768 patients. The patients in these studies were pathologically diagnosed with stomach cancer. Among these, 406 individuals received acupressure point stimulation treatment, while the remaining 362 were assigned to control groups. Eligibility criteria for enrollment in these studies were assessed, revealing that seven studies exclusively employed acupuncture as the therapeutic intervention for stomach cancer pain (22, 25, 26, 28, 30, 32). In these six papers, all acupuncture treatments used silver needles to puncture the relevant acupuncture points, rather than using newer acupuncture treatments such as electrical or thermal stimulation. One study used electroacupuncture as an intervention (24). Two studies implemented acupuncture injections for 63 patients (27, 29), and two studies employed moxibustion for addressing stomach cancer pain (23, 31). Within the control groups, seven studies adopted the World Health Organization’s recommended three-step analgesia approach (22, 23, 25, 26, 28, 30, 31). Two studies utilized Dumeraldine injections (27, 29), one study administered fentanyl (32), and another study employed conventional pain relief methods (24). Regarding the nature of stomach cancer pain, one study concentrated on postoperative pain following stomach cancer surgery (24), while a study looked at stomach cancer outbreak pain (28). The remaining studies pertained to chronic pain associated with stomach cancer.

**Table 1:** Characteristics of included studies.

Author	Country	Treatment group (males/females)	Control group (males/females)	Treatment group	Control group	Intervention duration and frequency	Inclusion outcome measures
Ban Niya·Baheti (22)	China	30 (17/13)	30 (22/8)	Acupuncture + Three-step Analgesia Method	Three-step Analgesia Method	Moxibustion for 20 min	NRS
Cao and Chen (23)	China	49 (27/22)	48 (23/25)	Moxibustion + Three-step Analgesia Method	Three-step Analgesia Method	Twice a day for 30 min	Analgesic effect
Gao (24)	China	30 (20/10)	30 (17/13)	Acupuncture + Three-step Analgesia Method	Three-step Analgesia Method	1 time daily for 30 min	NRS, Analgesic effect
Li et al. (25)	China	30 (16/14)	30 (13/17)	Acupuncture + Three-step Analgesia Method	Three-step Analgesia Method	Moxibustion for 30 min,1 time per day	Analgesic effect
Mi et al. (26)	China	32 (12/19)	30 (10/20)	Acupuncture + Three-step Analgesia Method	Three-step Analgesia Method	30 min each time, once every other day	Analgesic effect
Dou et al. (27)	China	43 (23/20)	38 (20/18)	Acupoint Injection	Intramuscular Injection	NA	Analgesic effect
Xia (28)	China	29 (25/4)	23 (16/7)	Acupuncture + Three-step Analgesia Method	Three-step Analgesia Method	NA	NRS, Analgesic effect
Zhan and Wan (29)	China	20 (NA)	20 (NA)	Acupoint Injection	Intramuscular Injection	NA	Analgesic effect
Zhang and Liu (30)	China	60 (44/16)	30 (18/12)	Acupuncture + Three-step Analgesia Method	Three-step Analgesia Method	2 times a day	Analgesic effect
Zou (31)	China	48 (26/22)	48 (24/24)	Moxibustion + Three-step Analgesia Method	Three-step Analgesia Method	2 times a day	Analgesic effect
Jiang et al. (32)	China	35 (17/18)	35 (16/19)	Acupuncture + Fentanyl	Fentanyl	1 time per day	NRS, Analgesic effect

### Quality assessment

**Figure 2:**
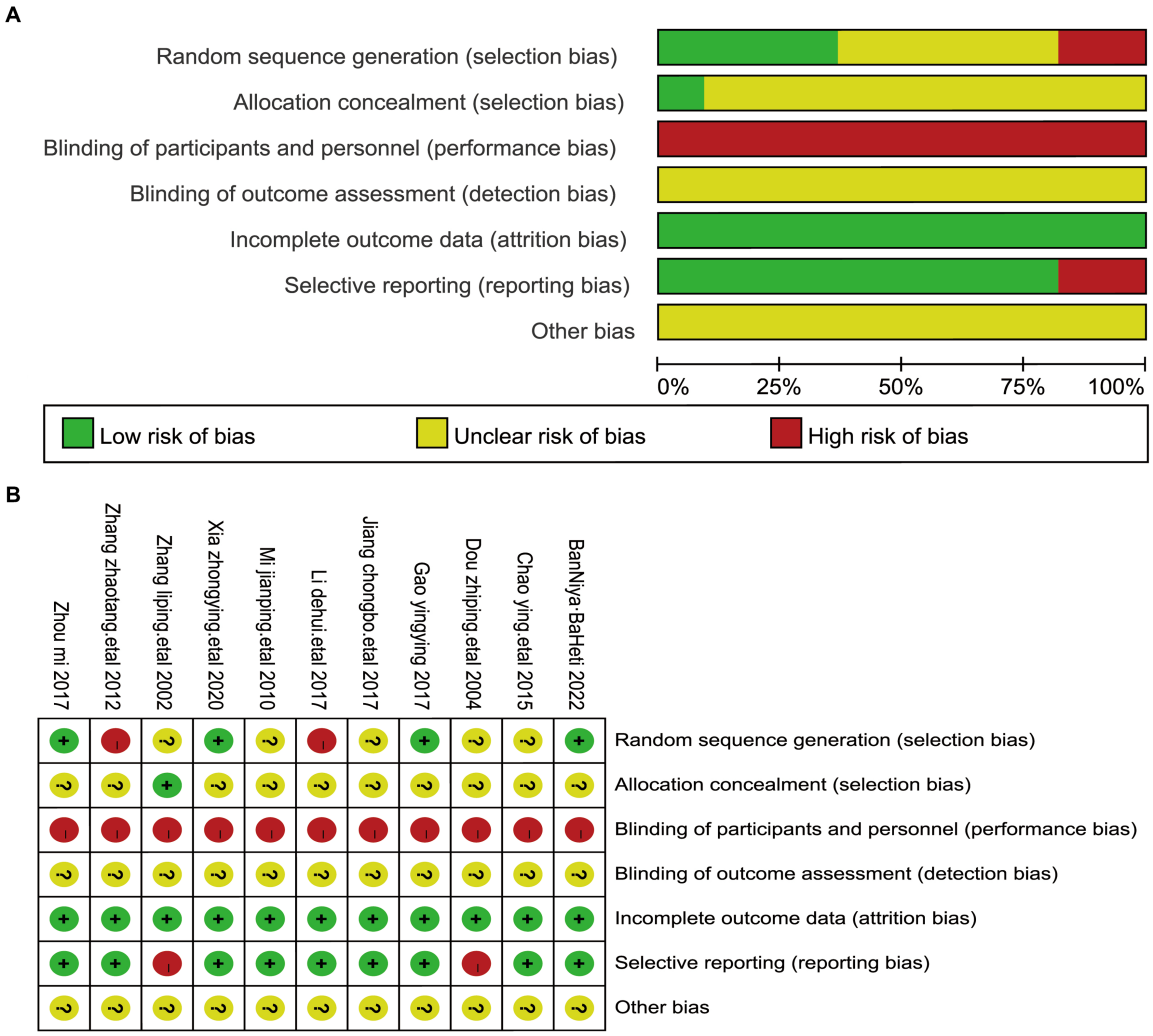
The figure represents the risk of bias assessment for the studies selected in the meta-analysis. **(A)** RoB graph: risk of bias graph—provides a visual summary of bias risk across studies, with green (low risk), yellow (unclear), and red (high risk) segments for bias categories. **(B)** RoB summary: risk of bias summary—details bias risk for each study in the meta-analysis, using colored circles (+ for low risk,? for unclear, and − for high risk) for bias categories.

The results of the methodological assessment are shown in Figure 2. Of the 11 studies that referred to random allocation methods, 4 were assessed as low risk because they used a table of random numbers (22, 24, 28, 31), 5 were categorized as unclear risk of bias because they provided insufficient information, and 2 were classified as high risk because they grouped patients in the order of their admission to the hospital (25, 30). None of the 11 studies described the allocation concealment process in sufficient detail to be judged as unclear risk of bias. In 11 studies, blinding of subjects or administrators was not possible because of significant differences in the use of acupuncture treatment between treatment and control groups (22–32). The completeness of the outcome data of all studies was judged to be at low risk of bias. Eleven studies were categorized as having a low risk of bias for selective reporting because all prespecified endpoints were reported and were rated as having a low risk of bias for selective reporting. Two studies (27, 29) were rated at high risk of bias for selective reporting because the endpoints were poorly reported. There were insufficient data to judge other risks of bias in 11 studies (22–32) (Table 2).

**Table 2:** Quality assessment of all the studies.

Studies	Characteristics of studies						
	Random sequence generation (selection bias)	Allocation concealment (selection bias)	Blinding of participants and personnel (performance bias)	Blinding of outcome assessment (detection bias)	Incomplete outcome data (attrition bias)	Selective reporting (reporting bias)	Other bias
Ban Niya·Baheti (22)	Low	Unclear	High	Unclear	Low	Low	Unclear
Cao and Chen (23)	Unclear	Unclear	High	Unclear	Low	Low	Unclear
Gao (24)	Low	Unclear	High	Unclear	Low	Low	Unclear
Li et al. (25)	High	Unclear	High	Unclear	Low	Low	Unclear
Mi et al. (26)	Unclear	Unclear	High	Unclear	Low	Low	Unclear
Dou et al. (27)	Unclear	Unclear	High	Unclear	Low	High	Unclear
Xia (28)	Low	Unclear	High	Unclear	Low	Low	Unclear
Zhan and Wan (29)	Unclear	Low	High	Unclear	Low	High	Unclear
Zhang and Liu (30)	High	Unclear	High	Unclear	Low	Low	Unclear
Zou (31)	Low	Unclear	High	Unclear	Low	Low	Unclear
Jiang et al. (32)	Unclear	Unclear	High	Unclear	Low	Low	Unclear

### Results of individual studies

#### Effects on NRS score of patients with stomach cancer pain

**Figure 3:**
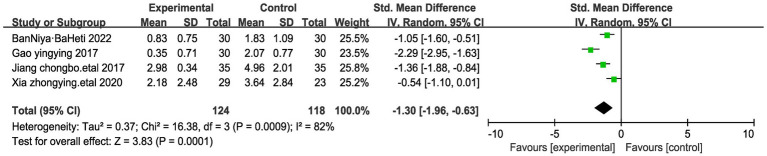
Forest plots of NRS score of stomach cancer pain after acupressure point stimulation treatment. The plot lists the included studies by the first author and publication year, showing the mean NRS scores for both experimental and control groups, along with their standard deviations and total number of participants. The weight of each study in the meta-analysis is indicated, reflecting its contribution to the overall effect size.

In four of the studies, NRS Scores were evaluated among a total of 242 participants (22, 24, 28, 32). NRS pain score uses the Numeric Rating Scale for Pain Levels to assess the pain level of the patient. Self-rating on a 10-point scale based on the degree, which is categorized into 1–10, allows for the classification of pain into different degrees based on the corresponding number, i.e., 0 for no pain, 1–3 for mild pain, 4–6 for moderate pain, and 7–10 for severe pain. A notable observation emerged wherein the NRS Scores were lower in cases where acupressure point stimulation treatment was administered. Given the substantial heterogeneity detected between these studies (I^2^ = 82%, *p* = 0.0009), a random-effects model was applied. The analysis demonstrated a statistically significant difference in NRS Scores (Standardized Mean Difference, SMD = −1.30, 95% CI −1.96 to −0.63, *p* < 0.001). We performed a sensitivity analysis of the results using the one-by-one exclusion method, and the results were statistically significant after arbitrarily excluding one study, indicating the robustness of the results (Table 3). This outcome suggests that the acupressure point stimulation treatment exhibited superior improvement within the treatment group compared to the control group receiving conventional pain relief methods (Figure 3).

**Table 3:** Sensitivity analysis of NRS showing pooled results after excluding one study.

Study of removal	Ban Niya·Baheti (22)	Gao (24)	Jiang et al. (32)	Xia (28)
SMD	−1.38 [−2.32, −0.45]	−0.99 [−1.46, −0.53]	−1.28 [−2.24, −0.32]	−1.54 [−2.22, −0.87]

#### Effects on significant efficacy rate of patients with stomach cancer pain

**Figure 4:**
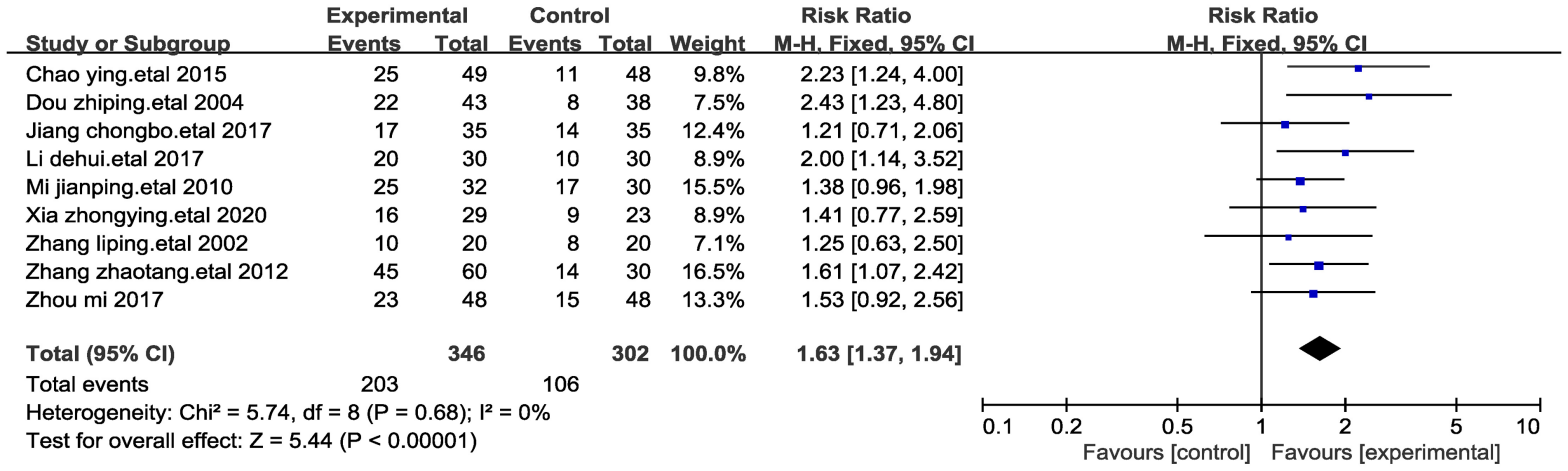
Forest plots of significantly efficient of stomach cancer pain after acupressure point stimulation treatment. The plot lists the included studies by the first author and publication year, showing the significantly efficient for both experimental and control groups, along with their risk ratio and total number of participants. The weight of each study in the meta-analysis is indicated, reflecting its contribution to the overall effect size.

According to the evaluation standard of pain relief effect formulated by WHO, the efficacy assessment is divided into 4 levels: (1) complete relief: the pain disappears, (2) effective relief: the pain is significantly reduced after taking the drug, which basically does not affect the patient’s normal sleep, (3) mild relief: the pain is significantly reduced after taking the drug, but it has a certain effect on normal sleep, and (4) ineffective: there is no relief of the pain after taking the drug. We define complete relief and effective relief as significant effective relief. A comprehensive meta-analysis encompassing nine studies, involving a total of 648 participants, was conducted to assess the significant efficacy rate (23, 25–32). The outcomes indicated a noteworthy increase in the significant efficacy rate within the acupressure point stimulation group in comparison to the control group (Relative Risk, RR = 1.63, 95% CI 1.37 to 1.94, *p* < 0.00001). We performed a sensitivity analysis of the results using the one-by-one exclusion method, and the results were statistically significant after arbitrarily excluding one study, indicating the robustness of the results (Table 4). Of significant note, there was no detectable heterogeneity among these studies (I^2^ = 0%, *p* = 0.68) (Figure 4).

**Table 4:** Sensitivity analysis of efficient showing pooled results after excluding one study.

Study of removal	Cao and Chen (23)	Dou et al. (27)	Jiang et al. (32)	Li et al. (25)	Mi et al. (26)	Xia (28)	Zhan and Wan (29)	Zhang and Liu (30)	Zou (31)	Gao (24)
SMD of significant efficacy rate	1.56 [1.30, 1.88]	1.56 [1.30, 1.87]	1.69 [1.40, 2.03]	1.59 [1.32, 1.91]	1.67 [1.38, 2.04]	1.65 [1.37, 1.98]	1.66 [1.38, 1.99]	1.63 [1.34, 1.98]	1.64 [1.36, 1.98]	NA
SMD of efficacy rate	1.14 [1.02, 1.28]	1.13 [1.02, 1.26]	1.19 [1.05, 1.34]	1.18 [1.04, 1.34]	1.20 [1.08, 1.34]	1.19 [1.04, 1.35]	1.17 [1.03, 1.33]	1.15 [1.02, 1.30]	1.17 [1.02, 1.33]	1.14 [1.03, 1.28]

#### Effects on efficacy rate of patients with stomach cancer pain

**Figure 5:**
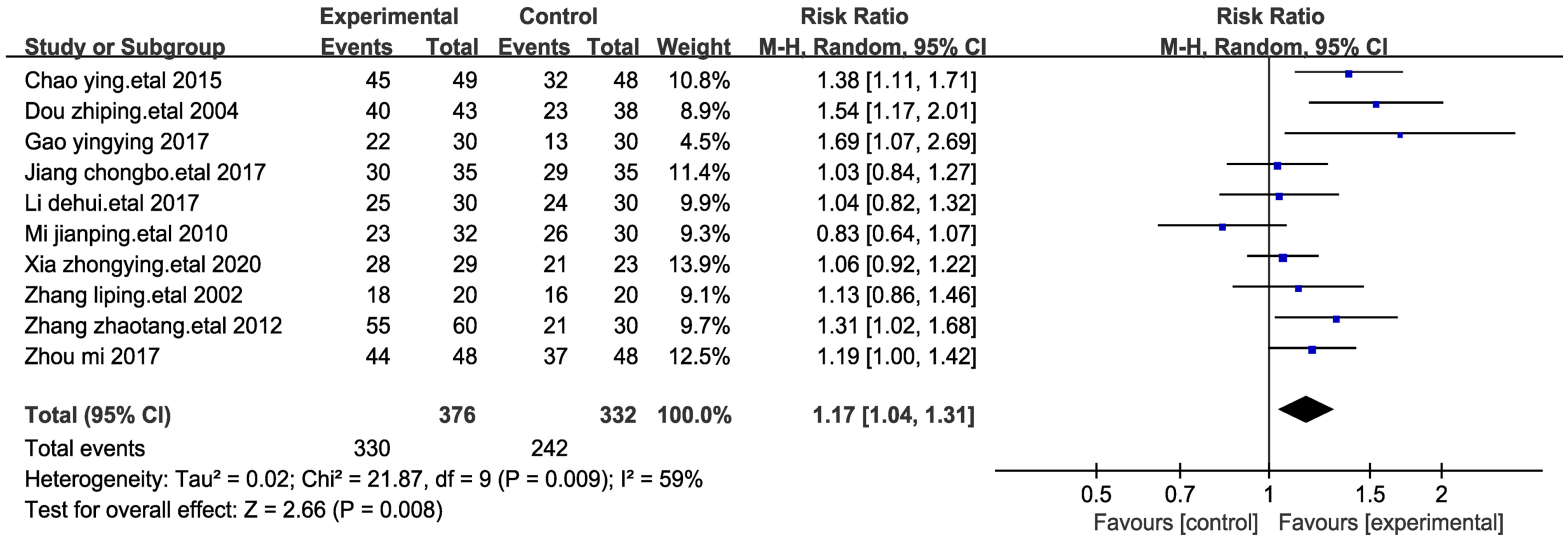
Forest plots of efficient of stomach cancer pain after acupressure point stimulation treatment. The plot lists the included studies by the first author and publication year, showing the efficient for both experimental and control groups, along with their risk ratio and total number of participants. The weight of each study in the meta-analysis is indicated, reflecting its contribution to the overall effect size.

A meta-analysis involving ten studies and encompassing a total of 704 participants was carried out to assess the significant efficacy rate (23–32). Given the observed high data heterogeneity between the two groups (I^2^ = 59%, *p* = 0.009), a random-effects model was employed for comparisons. We performed a sensitivity analysis of the results using the one-by-one exclusion method, and the results were statistically significant after arbitrarily excluding one study, indicating the robustness of the results (Table 4). The findings of our analysis revealed that, in comparison to the control group, acupressure point stimulation led to a notably higher rate of effective treatment (Relative Risk, RR = 1.17, 95% CI 1.04 to 1.31, *p* < 0.01) (Figure 5).

#### Adverse reactions

**Figure 6:**
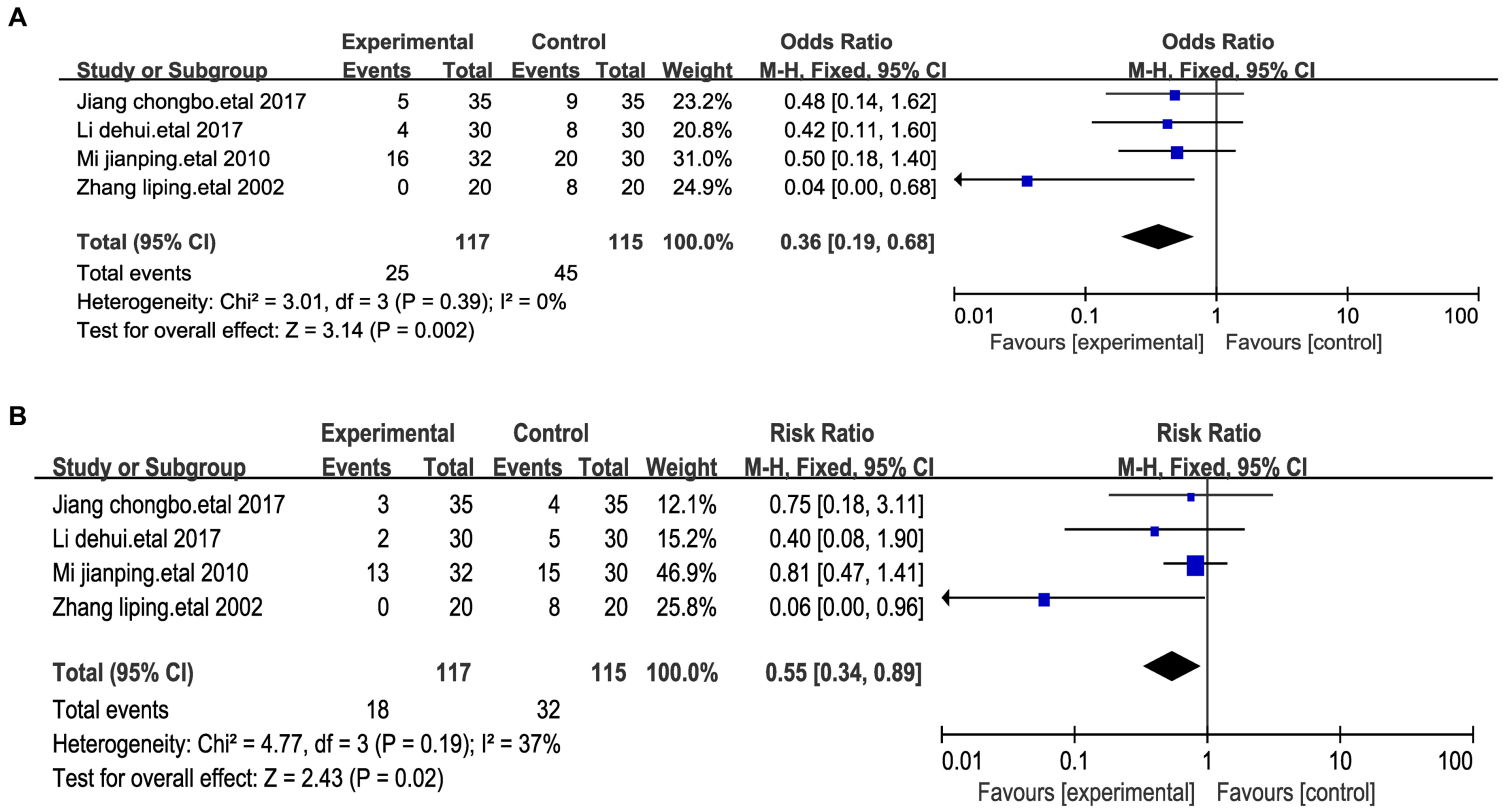
Forest plots of the adverse effects after treatment. **(A)** Forest maps of nausea. **(B)** Forest maps of vomiting.

A meta-analysis examining adverse reaction rates across four studies involving medication was conducted (25, 26, 29, 32). The primary adverse effects reported were nausea and vomiting. The pooled analysis of these four studies revealed that out of the 115 patients in the medication groups, 45 individuals experienced nausea (39.1%). In contrast, among the 117 patients receiving acupressure point stimulation, only 25 encountered nausea (21.3%) (Figure 6A). Similarly, across the four studies, 32 of the 115 patients in the medication groups experienced vomiting (27.8%), while only 18 of the 117 patients undergoing acupressure point stimulation therapy reported vomiting (15.3%) (Figure 6B). We performed a sensitivity analysis of the results using the one-by-one exclusion method, and the results were statistically significant after arbitrarily excluding one study, indicating that the results were robust. These findings collectively suggest that acupressure point stimulation therapy demonstrates a relatively higher level of safety when compared to the control group receiving medication.

### Sub-group analysis

#### Sub-group analysis of the effects on significant efficacy rate

**Figure 7:**
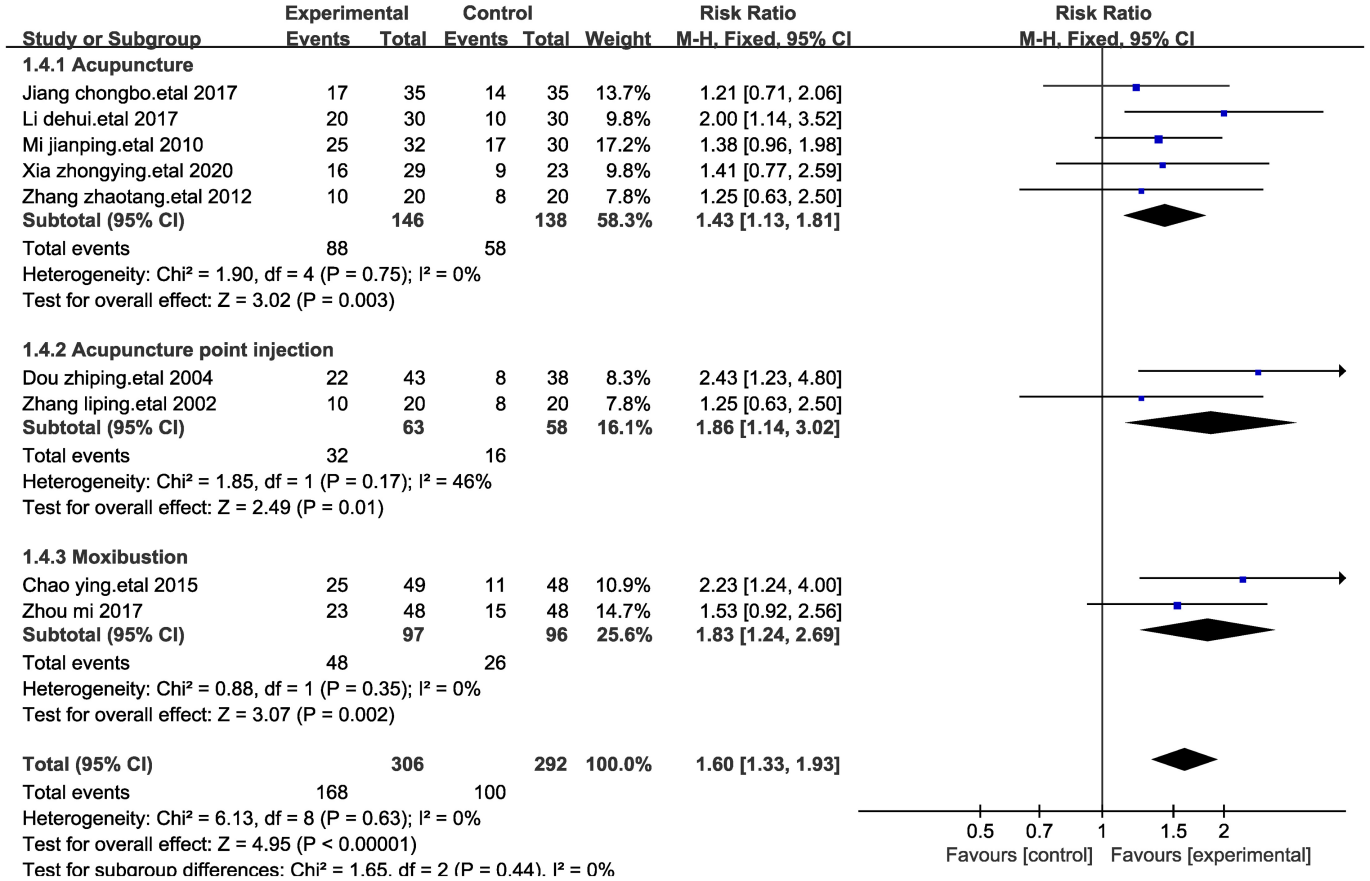
Forest plot for subgroup analysis with significant efficiency impact. This figure shows a forest plot of the different studies included in the meta-analysis assessing the effect of treatments such as acupuncture, acupoint injections and moxibustion on stomach cancer pain. Each study is listed by first author and year of publication, showing the total number of events in the trial and control groups and the corresponding weights. Risk Ratio (RR) and corresponding 95% confidence intervals (CI) provided a quantitative assessment of the effect size of each study.

During the sub-group meta-analysis of the included studies, a classification into three distinct subgroups was performed based on the diverse methods of stimulating acupuncture points in patients with stomach cancer pain. Within these subgroups, five studies employed acupuncture (25, 26, 32, 28, 30), two studies exclusively employed moxibustion (23, 31), and two studies utilized acupuncture point injections. Upon analyzing these three subgroups (27, 29), it was observed that the significant treatment response achieved through acupuncture injections (Relative Risk, RR = 1.86) surpassed that of acupuncture (RR = 1.43) and moxibustion (RR = 1.83) (Figure 7). This pattern could suggest the potential superiority of acupuncture points in treating stomach cancer pain.

#### Sub-group analysis of the effects on efficacy rate

We categorized the studies into three subgroups based on the distinct methods of stimulating acupuncture points in patients with stomach cancer pain. Among these, five studies employed acupuncture (25, 26, 32, 28, 30), two studies solely focused on moxibustion (23, 31), and two studies utilized acupuncture point injections (27, 29).

**Figure 8:**
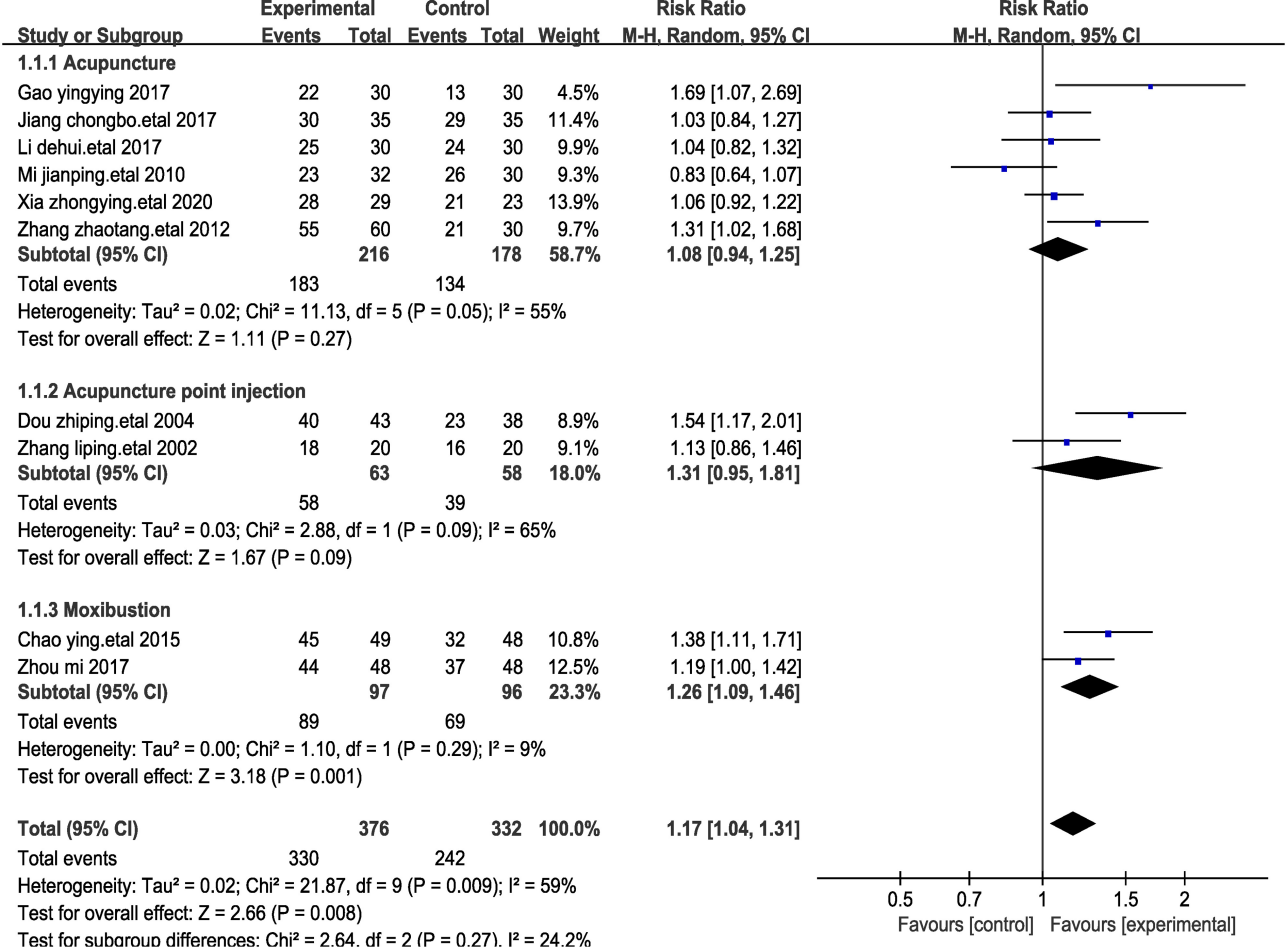
Forest plot for subgroup analysis with efficiency impact. This figure shows a forest plot of the different studies included in the meta-analysis assessing the effect of treatments such as acupuncture, acupoint injections and moxibustion on stomach cancer pain. Each study is listed by first author and year of publication, showing the total number of events in the trial and control groups and the corresponding weights. Risk Ratio (RR) and corresponding 95% confidence intervals (CI) provided a quantitative assessment of the effect size of each study.

In these three subgroups, the notable treatment response achieved through acupuncture injections (Relative Risk, RR = 1.31) surpassed those of acupuncture (RR = 1.08) and moxibustion (RR = 1.26) (Figure 8). Once again, these findings reinforce the positive outcomes associated with acupuncture across various acupuncture point stimulation techniques.

#### Analysis of the frequency of acupuncture point use

**Figure 9:**
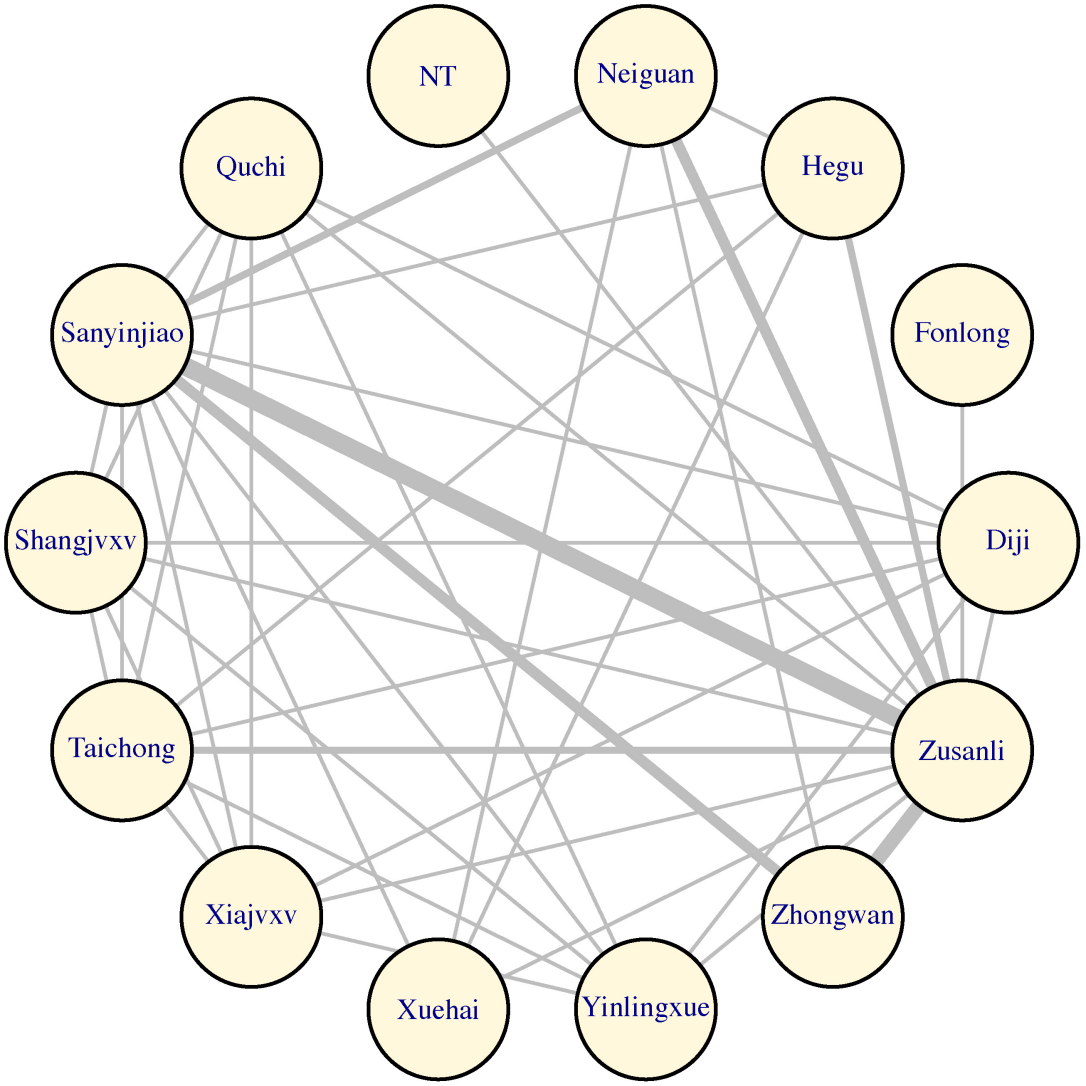
The reticulation that occurs at different acupoints during treatment. This figure represents the network relationship between different acupuncture points. Each circle represents a specific acupuncture point, e.g., “Neiguan” or “Zusanli,” and the lines between them indicate interactions or correlations. The thickness of the lines may represent differences in the strength of the association, but this is not explicitly stated in the figure notes. The overall network diagram provides a visual way to understand and analyze the possible synergistic effect or combination of different acupuncture points in treatment.

We conducted a comprehensive analysis of all the acupuncture points employed in acupuncture point stimulation. Utilizing the GEMTC package in R version 4.3.0, we determined the frequency of usage for different acupuncture points. The graphical representation displayed a mesh diagram, highlighting the prominent utilization of certain acupuncture points. Specifically, the Sanyinjiao and Zusanli acupuncture points were the most frequently used. Additionally, the Zhongguan and Neiguan acupuncture points also saw widespread application. This outcome holds valuable implications for future acupoint treatments targeting stomach cancer pain, assisting medical practitioners in making informed choices regarding acupuncture point selection for treatment (Figure 9).

#### Publishing bias

We performed a funnel plot analysis of the literature on the use of acupressure point stimulation and medication. The results showed that multiple funnel plots were asymmetric, indicating publication bias (Supplementary Figure S1).

## Discussion

Before treating pain, traditional Chinese medicine such as acupuncture had been widely used to treat a wide range of ailments, and it has shown good results in different aspects. For example, within the realms of diabetes and neurological disorders, acupuncture exhibits remarkable advantages (33–36). Network meta-analyses have previously explored traditional Chinese medicine interventions, including acupuncture (37). In 2023, a scoping review of systematic reviews and meta-analyses was conducted to assess the effectiveness of acupuncture as a treatment for cancer-related pain (38). In the context of the scoping review, a total of 25 systematic reviews focusing on acupuncture for cancer pain management were incorporated. These reviews encompassed a diverse array of primary studies characterized by varying study designs and qualities. The review confirmed the efficacy of acupuncture in mitigating cancer-related pain. However, traditional Chinese medicine treatments such as moxibustion have often been neglected in the realm of cancer pain research, and pain associated with different types of cancers has rarely been examined separately. Recognizing this gap in the existing literature, we decision to conduct this meta-analysis holds significance. Through this meta-analysis, we aimed to thoroughly analyze the therapeutic effectiveness of acupuncture point stimulation specifically in the context of stomach cancer pain. This initiative contributes to filling the research void and providing valuable insights into the potential benefits of acupressure point stimulation for individuals dealing with stomach cancer pain. Acupuncture points constitute a cornerstone within the acupuncture theory of Oriental medicine, along with meridian pathways (39). Extensive endeavors have been directed towards unraveling the scientific underpinnings that govern the functions of acupuncture points and meridian pulses. For example, the characteristics of acupuncture points and meridian pulses, including electrical (40), temperature (41), anatomical (42), and photon (43) characteristics, have been identified. In addition to the direct characteristics of acupuncture points, several studies using bioimaging technology, such as electroencephalography (EEG) and functional magnetic resonance imaging, have evaluated the effects of the physical stimulation of acupuncture points on the human body (44–46). Indeed, acupuncture points also serve a role in analgesia. A previous study showcased the efficacy of acupuncture point stimulation in achieving analgesic effects in the context of lumbar spine pain (47).

The findings from this meta-analysis strongly indicate that acupressure point stimulation treatment holds a greater therapeutic efficacy compared to pharmacotherapy administered in isolation. This conclusion is substantiated by the observed reduction in NRS scores and the higher treatment efficiency evident within the acupressure point stimulation group. Importantly, it should be highlighted that none of the included studies reported any serious adverse events among patients subjected to acupressure point stimulation treatment. Furthermore, acupressure point stimulation treatment demonstrated improved outcomes even in terms of adverse reactions. This aligns with earlier research that has also highlighted the efficacy of acupressure point stimulation in mitigating adverse effects (48). Subgroup analyses further suggest that acupoint injections yield more favorable treatment outcomes.

Nevertheless, it’s important to acknowledge that the considerable heterogeneity across the included studies poses a constraint on drawing definitive conclusions. Despite the promising outcomes, our study is not without limitations. For instance, studies with small sample sizes were incorporated, and certain studies were deficient in terms of available data. Furthermore, some studies lacked clear and comprehensive details. And due to study limitations, the outcome metrics of the included studies were not abundant, limitations that highlight the current lack of comprehensive clinical trials in this area. These limitations highlight the pressing necessity for additional well-designed randomized controlled trials with larger patient cohorts. Bridging the evident gap between preclinical research and clinical studies requires a concerted effort to generate more robust evidence and enhance our understanding of the potential benefits of acupressure point stimulation treatment.

## Conclusion

To our knowledge, this is the first study dedicated to evaluating the clinical efficacy of acupressure point stimulation in the treatment of patients with stomach cancer pain. In essence, acupressure point stimulation demonstrated significant improvements in safety, significantly efficient ratio, efficient ratio, and reduced the NRS score in patients with stomach cancer pain. Nevertheless, these promising observations are based on a limited number of studies with relatively small cohorts, necessitating further large, well-designed clinical trials for confirmation. Undoubtedly, more discoveries related to the mechanism of therapeutic action will be revealed in the future, as well as ways to maximize the benefits of acupressure point stimulation therapy.

## Data availability statement

The original contributions presented in the study are included in the article/Supplementary material, further inquiries can be directed to the corresponding authors.

## Author contributions

XZ: Conceptualization, Data curation, Formal analysis, Writing – original draft. JZ: Conceptualization, Data curation, Writing – original draft. LJ: Methodology, Writing – original draft. SZ: Visualization, Writing – original draft. YG: Project administration, Writing – original draft. JT: Conceptualization, Writing – original draft. TP: Data curation, Writing – original draft. XQ: Visualization, Writing – original draft. HC: Project administration, Resources, Supervision, Writing – original draft, Writing – review & editing. SH: Funding acquisition, Project administration, Supervision, Writing – original draft, Writing – review & editing.
