# Supplementary material for: Therapeutic efficacy of acupuncture point stimulation for stomach cancer pain: a systematic review and meta-analysis
Source: Front Neurol. 2024 Apr 4;15:1334657. doi: 10.3389/fneur.2024.1334657 (PMC11024429; doi:10.3389/fneur.2024.1334657)
Supplement: Supplementary file 1 [file Data_Sheet_1.DOCX]

**PubMed：**

1. **(((((((gastric cancer[Title/Abstract]) OR (Neoplasm, Stomach[Title/Abstract])) OR (Gastric Neoplasms[Title/Abstract])) OR (Neoplasm, Gastric[Title/Abstract])) OR (Cancer of Stomach[Title/Abstract])) OR (Stomach Cancers[Title/Abstract])) OR (Cancer, Gastric[Title/Abstract])) OR (Gastric Cancer, Familial Diffuse[Title/Abstract])**
2. **(((((((Acupressure point stimulation[Title/Abstract]) OR (Acupressure point therapy[Title/Abstract])) OR (acupuncture[Title/Abstract])) OR (Acupuncture[Title/Abstract])) OR (Electroacupuncture[Title/Abstract])) OR (Fire needle[Title/Abstract])) OR (Acupuncture point injection[Title/Abstract])) OR (Acupressure points[Title/Abstract])**
3. **((((((pain[Title/Abstract]) OR (Pain, Burning[Title/Abstract])) OR (Suffering, Physical[Title/Abstract])) OR (Physical Suffering[Title/Abstract])) OR (Pain, Migratory[Title/Abstract])) OR (Pain, Radiating[Title/Abstract])) OR (Ache[Title/Abstract])**
4. **((((((((pain[Title/Abstract]) OR (Pain, Burning[Title/Abstract])) OR (Suffering, Physical[Title/Abstract])) OR (Physical Suffering[Title/Abstract])) OR (Pain, Migratory[Title/Abstract])) OR (Pain, Radiating[Title/Abstract])) OR (Ache[Title/Abstract])) AND ((((((((Acupressure point stimulation[Title/Abstract]) OR (Acupressure point therapy[Title/Abstract])) OR (acupuncture[Title/Abstract])) OR (Acupuncture[Title/Abstract])) OR (Electroacupuncture[Title/Abstract])) OR (Fire needle[Title/Abstract])) OR (Acupuncture point injection[Title/Abstract])) OR (Acupressure points[Title/Abstract]))) AND ((((((((gastric cancer[Title/Abstract]) OR (Neoplasm, Stomach[Title/Abstract])) OR (Gastric Neoplasms[Title/Abstract])) OR (Neoplasm, Gastric[Title/Abstract])) OR (Cancer of Stomach[Title/Abstract])) OR (Stomach Cancers[Title/Abstract])) OR (Cancer, Gastric[Title/Abstract])) OR (Gastric Cancer, Familial Diffuse[Title/Abstract]))**

Wos

1. **(((((((TS=(gastric cancer)) OR TS=(Neoplasm, Stomach)) OR TS=(Gastric Neoplasms)) OR TS=(Neoplasm, Gastric)) OR TS=(Cancer of Stomach)) OR TS=(Stomach Cancers)) OR TS=(Cancer, Gastric)) OR TS=(Gastric Cancer, Familial Diffuse)**
2. **(((((((TS=(Acupressure point stimulation)) OR TS=(Acupressure point therapy)) OR TS=(acupuncture)) OR TS=(Acupuncture)) OR TS=(Electroacupuncture)) OR TS=(Fire needle)) OR TS=(Acupuncture point injection)) OR TS=(Acupressure points)**
3. **((((((TS=(pain)) OR TS=(Pain, Burning)) OR TS=(Suffering, Physical)) OR TS=(Physical Suffering)) OR TS=(Pain, Migratory)) OR TS=(Pain, Radiating)) OR TS=(Ache)**
4. **#1 AND #2 AND #3**

Cochrane

1. ‘gastric cancer’ OR ‘Neoplasm, Stomach’ OR ‘Gastric Neoplasms’ OR ‘Neoplasm, Gastric’ OR ‘Cancer of Stomach’ OR ‘Stomach Cancers’ OR ‘Cancer, Gastric’ OR ‘Gastric Cancer, Familial Diffuse’
2. ‘Acupressure point stimulation’ OR ‘Acupressure point therapy’ OR ‘acupuncture’ OR ‘Acupuncture’ OR ‘Electroacupuncture’ OR ‘Fire needle’ OR ‘Acupuncture point injection’ OR ‘Acupressure points’
3. ‘pain’ OR ‘Pain, Burning’ OR ‘Suffering, Physical’ OR ‘Physical Suffering’ OR ‘Pain, Migratory’ OR ‘Pain, Radiating’ OR ‘Ache’
4. ‘gastric cancer’ OR ‘Neoplasm, Stomach’ OR ‘Gastric Neoplasms’ OR ‘Neoplasm, Gastric’ OR ‘Cancer of Stomach’ OR ‘Stomach Cancers’ OR ‘Cancer, Gastric’ OR ‘Gastric Cancer, Familial Diffuse’ in Title Abstract Keyword AND ‘Acupressure point stimulation’ OR ‘Acupressure point therapy’ OR ‘acupuncture’ OR ‘Acupuncture’ OR ‘Electroacupuncture’ OR ‘Fire needle’ OR ‘Acupuncture point injection’ OR ‘Acupressure points’ in Title Abstract Keyword AND ‘pain’ OR ‘Pain, Burning’ OR ‘Suffering, Physical’ OR ‘Physical Suffering’ OR ‘Pain, Migratory’ OR ‘Pain, Radiating’ OR ‘Ache’ in Title Abstract Keyword - (Word variations have been searched)

Embase

1. 'pain'/exp OR 'pain' OR 'pain, burning' OR 'suffering, physical' OR 'physical suffering' OR 'pain, migratory' OR 'pain, radiating' OR 'ache'

2. 'acupressure point stimulation' OR 'acupressure point therapy' OR 'acupuncture'/exp OR 'acupuncture' OR 'electroacupuncture'/exp OR 'electroacupuncture' OR 'fire needle' OR 'acupuncture point injection' OR 'acupressure points'

3. 'gastric cancer'/exp OR 'gastric cancer' OR 'neoplasm, stomach' OR 'gastric neoplasms' OR 'neoplasm, gastric' OR 'cancer of stomach' OR 'stomach cancers' OR 'cancer, gastric' OR 'gastric cancer, familial diffuse'

4. #1 AND #2 AND #3

Wanfang

主题：（（“针灸”or “针刺”or “火针”or “电针”or “穴位刺激”or “穴位治疗”or “穴位注射”or “艾灸”or “穴位按压”）and（“胃癌”or “肿瘤”or “胃肿瘤”or “癌症”or “胃切除术”or “胃癌症”or “胃癌痛”or “胃手术”）and（“疼痛”or “不适”or “隐痛”or “剧痛”or “钝痛”or “刺痛”or “酸痛”or “灼痛”or “痛感”））

Vip

1.针灸+针刺+火针+电针+穴位刺激+穴位治疗+穴位注射+艾灸 +穴位按压

2.胃癌+肿瘤+胃肿瘤+癌症+胃切除术+胃癌症+胃癌痛+胃手术

3.疼痛+不适+隐痛+剧痛+钝痛+刺痛+酸痛+灼痛+痛感

4.#1+#2+#3

Cnki

1.针灸+针刺+火针+电针+穴位刺激+穴位治疗+穴位注射+艾灸 +穴位按压

2.胃癌+肿瘤+胃肿瘤+癌症+胃切除术+胃癌症+胃癌痛+胃手术

3.疼痛+不适+隐痛+剧痛+钝痛+刺痛+酸痛+灼痛+痛感

4. #1+#2+#3
