# Supplementary material for: Therapeutic efficacy of acupuncture point stimulation for stomach cancer pain: a systematic review and meta-analysis
Source: Front Neurol. 2024 Apr 4;15:1334657. doi: 10.3389/fneur.2024.1334657 (PMC11024429; doi:10.3389/fneur.2024.1334657)
Supplement: Supplementary file 2 [file Table_1.DOCX]

| Author | Country | Treatment group(Males/Females) | Control group(Males/Females) | Treatment group | Control group | Intervention Duration and Frequency | Inclusion Outcome Measures |
| --- | --- | --- | --- | --- | --- | --- | --- |
| BanNiya·BaHeti,2022 | China | 30(17/13) | 30(22/8) | Acupuncture + Three-step Analgesia Method | Three-step Analgesia Method | Moxibustion for 20 min | NRS |
| Chao ying.etal,2015 | China | 49(27/22) | 48(23/25) | Moxibustion + Three-step Analgesia Method | Three-step Analgesia Method | Twice a day for 30 minutes | Analgesic effect |
| Gao yingying,2017 | China | 30(20/10) | 30(17/13) | Acupuncture + Three-step Analgesia Method | Three-step Analgesia Method | 1 time daily for 30 minutes | NRS ,Analgesic effect |
| Li dehui.etal,2017 | China | 30(16/14) | 30(13/17) | Acupuncture + Three-step Analgesia Method | Three-step Analgesia Method | Moxibustion for 30 min,1 time per day | Analgesic effect |
| Mi jianping.etal,2010 | China | 32(12/19) | 30(10/20) | Acupuncture + Three-step Analgesia Method | Three-step Analgesia Method | 30 minutes each time, once every other day | Analgesic effect |
| Dou zhiping.etal,2004 | China | 43(23/20) | 38(20/18) | Acupoint Injection | Intramuscular Injection | NA | Analgesic effect |
| Xia zhongying.etal,2020 | China | 29(25/4) | 23(16/7) | Acupuncture + Three-step Analgesia Method | Three-step Analgesia Method | NA | NRS ,Analgesic effect |
| Zhang liping.etal,2002 | China | 20(NA) | 20(NA) | Acupoint Injection | Intramuscular Injection | NA | Analgesic effect |
| Zhang zhaotang.etal,2012 | China | 60(44/16) | 30(18/12) | Acupuncture + Three-step Analgesia Method | Three-step Analgesia Method | 2 times a day | Analgesic effect |
| Zhou mi,2017 | China | 48(26/22) | 48(24/24) | Moxibustion + Three-step Analgesia Method | Three-step Analgesia Method | 2 times a day | Analgesic effect |
| Jiang chongbo.etal,2017 | China | 35(17/18) | 35(16/19) | Acupuncture + Fentanyl | Fentanyl | 1 time per day | NRS ,Analgesic effect |
