# Supplementary material for: Therapeutic efficacy of acupuncture point stimulation for stomach cancer pain: a systematic review and meta-analysis
Source: Front Neurol. 2024 Apr 4;15:1334657. doi: 10.3389/fneur.2024.1334657 (PMC11024429; doi:10.3389/fneur.2024.1334657)
Supplement: Supplementary file 3 [file Table_2.DOCX]

| Studies | Characteristics of studies | | | | | | |
| --- | --- | --- | --- | --- | --- | --- | --- |
|  | Random sequence generation (selection bias) | Allocation concealment (selection bias) | Blinding of participants and personnel (performance bias) | Blinding of outcome assessment (detection bias) | Incomplete outcome data (attrition bias) | Selective reporting (reporting bias) | Other bias |
| BanNiya·BaHeti,2022 | low | unclear | high | unclear | low | low | unclear |
| Chao ying.etal,2015 | unclear | unclear | high | unclear | low | low | unclear |
| Gao yingying,2017 | low | unclear | high | unclear | low | low | unclear |
| Li dehui.etal,2017 | high | unclear | high | unclear | low | low | unclear |
| Mi jianping.etal,2010 | unclear | unclear | high | unclear | low | low | unclear |
| Dou zhiping.etal,2004 | unclear | unclear | high | unclear | low | high | unclear |
| Xia zhongying.etal,2020 | low | unclear | high | unclear | low | low | unclear |
| Zhang liping.etal,2002 | unclear | low | high | unclear | low | high | unclear |
| Zhang zhaotang.etal,2012 | high | unclear | high | unclear | low | low | unclear |
| Zhou mi,2017 | low | unclear | high | unclear | low | low | unclear |
| Jiang chongbo.etal,2017 | unclear | unclear | high | unclear | low | low | unclear |
