# Supplementary material for: Therapeutic efficacy of acupuncture point stimulation for stomach cancer pain: a systematic review and meta-analysis
Source: Front Neurol. 2024 Apr 4;15:1334657. doi: 10.3389/fneur.2024.1334657 (PMC11024429; doi:10.3389/fneur.2024.1334657)
Supplement: Supplementary file 4 [file Table_3.DOCX]

| Study of Removal | BanNiya·BaHeti 2022 | Gao yingying 2017 | Jiang chongbo.etal 2017 | Xia zhongying.etal 2020 |
| --- | --- | --- | --- | --- |
| SMD | -1.38 [-2.32, -0.45] | -0.99 [-1.46, -0.53] | -1.28 [-2.24, -0.32] | -1.54 [-2.22, -0.87] |
