# Supplementary material for: Therapeutic efficacy of acupuncture point stimulation for stomach cancer pain: a systematic review and meta-analysis
Source: Front Neurol. 2024 Apr 4;15:1334657. doi: 10.3389/fneur.2024.1334657 (PMC11024429; doi:10.3389/fneur.2024.1334657)
Supplement: Supplementary file 5 [file Table_4.DOCX]

| Study of Removal | Chao ying.etal 2015 | Dou zhiping.etal 2004 | Jiang chongbo.etal 2017 | Li dehui.etal 2017 | Mi jianping.etal 2010 | Xia zhongying.etal 2020 | Zhang liping.etal 2002 | Zhang zhaotang.etal 2012 | Zhou mi 2017 | Gao yingying 2017 |
| --- | --- | --- | --- | --- | --- | --- | --- | --- | --- | --- |
| SMD of significant efficacy rate | 1.56 [1.30, 1.88] | 1.56 [1.30, 1.87] | 1.69 [1.40, 2.03] | 1.59 [1.32, 1.91] | 1.67 [1.38, 2.04] | 1.65 [1.37, 1.98] | 1.66 [1.38, 1.99] | 1.63 [1.34, 1.98] | 1.64 [1.36, 1.98] | NA |
| SMD of efficacy rate | 1.14 [1.02, 1.28] | 1.13 [1.02, 1.26] | 1.19 [1.05, 1.34] | 1.18 [1.04, 1.34] | 1.20 [1.08, 1.34] | 1.19 [1.04, 1.35] | 1.17 [1.03, 1.33] | 1.15 [1.02, 1.30] | 1.17 [1.02, 1.33] | 1.14 [1.03, 1.28] |
