# Supplementary figures and images for: Therapeutic efficacy of acupuncture point stimulation for stomach cancer pain: a systematic review and meta-analysis
Source: Front Neurol. 2024 Apr 4;15:1334657. doi: 10.3389/fneur.2024.1334657 (PMC11024429; doi:10.3389/fneur.2024.1334657)

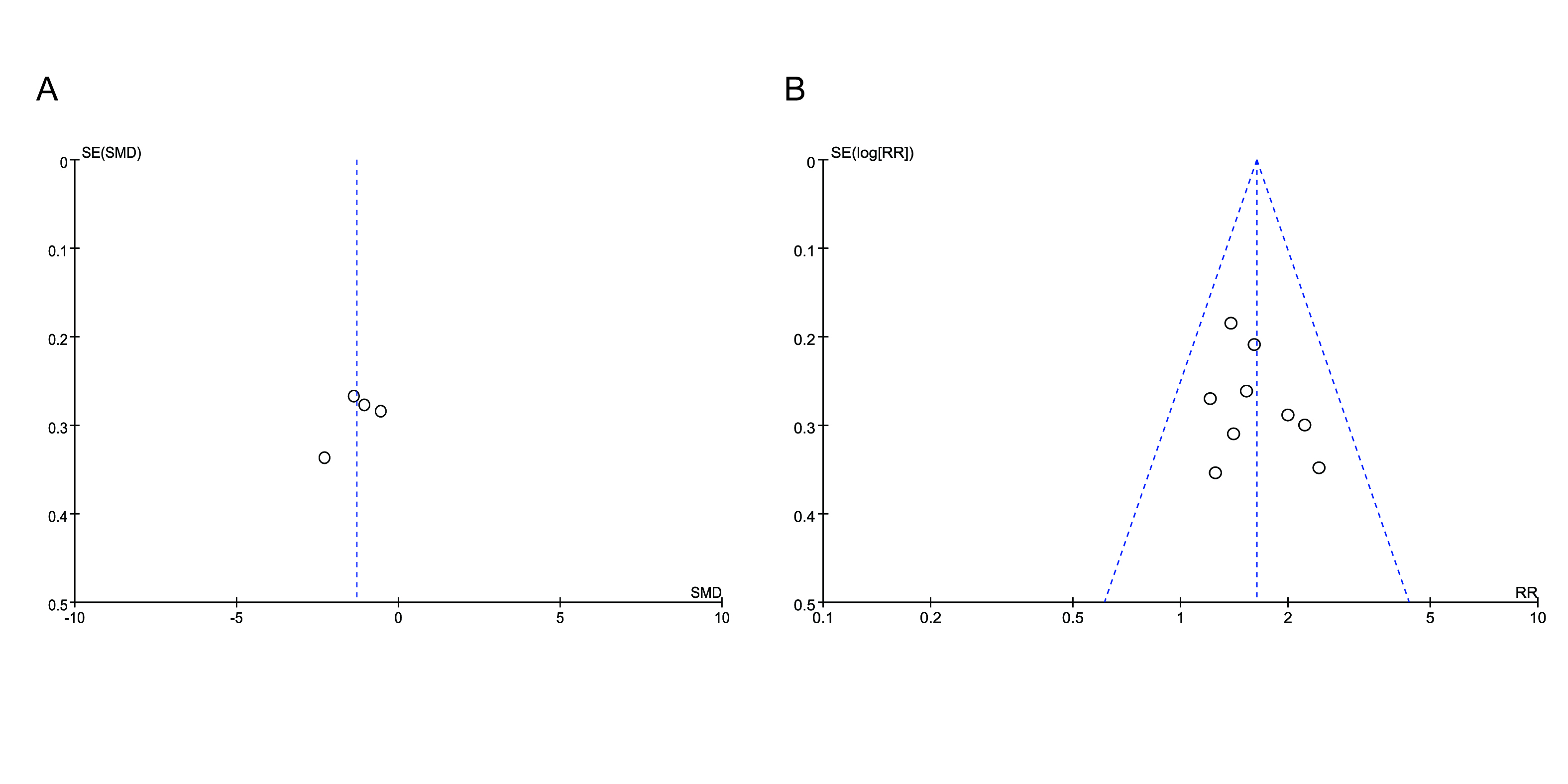

Supplement: Supplementary file 6 [file Image_1.JPEG]
